# Clinically Relevant Solution for the Hypothermic Storage and Transportation of Human Multipotent Mesenchymal Stromal Cells

**DOI:** 10.1155/2019/5909524

**Published:** 2019-01-20

**Authors:** Yuriy Petrenko, Milada Chudickova, Irena Vackova, Tomas Groh, Eliska Kosnarova, Jitka Cejkova, Karolina Turnovcova, Alexander Petrenko, Eva Sykova, Sarka Kubinova

**Affiliations:** ^1^Institute of Experimental Medicine of the Czech Academy of Sciences, Prague, Czech Republic; ^2^Bioinova Ltd., Prague, Czech Republic; ^3^Institute for Problems of Cryobiology and Cryomedicine, National Academy of Sciences of Ukraine, Kharkiv, Ukraine; ^4^Institute of Neuroimmunology, Slovak Academy of Sciences, Bratislava, Slovakia

## Abstract

The wide use of human multipotent mesenchymal stromal cells (MSCs) in clinical trials requires a full-scale safety and identity evaluation of the cellular product and subsequent transportation between research/medical centres. This necessitates the prolonged hypothermic storage of cells prior to application. The development of new, nontoxic, and efficient media, providing high viability and well-preserved therapeutic properties of MSCs during hypothermic storage, is highly relevant for a successful clinical outcome. In this study, a simple and effective trehalose-based solution was developed for the hypothermic storage of human bone marrow MSC suspensions for further clinical applications. Human bone marrow MSCs were stored at 4°C for 24, 48, and 72 hrs in the developed buffered trehalose solution and compared to several research and clinical grade media: Plasma-Lyte® 148, HypoThermosol® FRS, and Ringer's solution. After the storage, the preservation of viability, identity, and therapeutically associated properties of MSCs were assessed. The hypothermic storage of MSCs in the new buffered trehalose solution provided significantly higher MSC recovery rates and ability of cells for attachment and further proliferation, compared to Plasma-Lyte® 148 and Ringer's solution, and was comparable to research-grade HypoThermosol® FRS. There were no differences in the immunophenotype, osteogenic, and adipogenic differentiation and the immunomodulatory properties of MSCs after 72 hrs of cold storage in these solutions. The obtained results together with the confirmed therapeutic properties of trehalose previously described provide sufficient evidence that the developed trehalose medium can be applied as a low-cost and efficient solution for the hypothermic storage of MSC suspensions, with a high potential for translation into clinical practice.

## 1. Introduction

Multipotent mesenchymal stromal cells (MSCs) are currently the most widely studied and applied cell type in regenerative medicine, with more than 700 registered clinical trials conducted worldwide (https://clinicaltrials.gov). High cell accessibility together with their unique paracrine and replacement properties drives the MSC research towards its translation into clinical practice [1, 2]. The clinical grade manufacturing process of MSCs comprises a number of regulated steps, aimed at assuring the safety and identity of the final cellular product. Many studies show different aspects of the clinical grade manufacturing of MSCs [3, 4]. The application of a chemically defined culture media, xeno-free supplements for in vitro expansion, and banking of MSCs are thoroughly discussed elsewhere [5, 6]. These studies provide key information for optimising the cell manufacturing process, to obtain safe and efficient cellular therapies.

Prior to clinical application, the safety and identity of the final MSC-based product must be confirmed by a panel of techniques, recommended by the regulatory authorities. The absence of viral and microbial contamination, which may occur during isolation and in vitro cell expansion, represents the main aspect, confirming the safety of MSCs for clinical use. The microbiological quality control procedures must be defined and validated in each laboratory, according to the European Medicinal Agency guidelines and pharmacopoeia rules for parenteral medicinal products [7, 8]. Wherein, the final determination of sterility should be prepared prior to packaging (e.g., in the finished product), but in the event that the product would undergo cryopreservation, reseeding after thawing or any further manipulation requires repeated sterility testing [9]. Unfortunately, the fastest tests that are currently available require up to 48-72 hrs to obtain the results and any data obtained earlier are considered preliminary and insufficient. The Food and Drug Administration (FDA) has recently reported the suitability of several rapid tests for the fast (up to 72 hrs) detection of microorganisms in liquid media: BacT/ALERT® (bioMérieux), BACTEC™ (Becton Dickinson), and Rapid Milliflex® from Merck Millipore [10]. However, even though rapid systems for contamination assessment are already available, such prolonged storage of cells, for 48-72 hrs in inappropriate conditions, would impact their viability and functional properties. Therefore, the search for optimal, reproducible storage conditions represents a critical issue in the cell manufacturing process, which lacks due consideration.

The hypothermic preservation in a nonfrozen state (at 2-8°C) represents the most reproducible and easy storage system; however, in incorrect conditions, it would lead to irreversible changes in cellular structure, leading to cell apoptosis and death. It is known that hypothermia causes the perturbation of ion balances, notably mono- and divalent cations (Na^+^, K^+^, and Ca^2+^) and anions (Cl^−^), which leads to osmotic cell swelling, oxidative stress, and an extracellular and intracellular pH switch [11, 12]. Thus, in ideal conditions, the vehicle solution for MSC administration should provide protection against these damaging factors. Conventional vehicle solutions for cell delivery in clinical settings usually represent either a simple physiological saline (e.g., Ringer's solution for injections or 0.9% sodium chloride) or more complex electrolyte solutions (e.g., Plasma-Lyte® 148 or similar), which additionally may contain either human serum albumin or other nonionic plasma volume expanders (hydroxyethyl starch or dextran) [2]. However, the question regarding the applicability of such solutes for the hypothermic preservation of MSCs for 48 hrs–72 hrs should still be the subject of extensive research.

The application of specialised solutions developed for hypothermic preservation may represent an alternative way of optimising the storage conditions of cell-based products. Several widely used, commercially available solutions have been confirmed as effective for maintaining the viability of different cell types during cold storage [11, 13–16]. The University of Wisconsin Solution (UW solution) is considered as a gold standard medium for whole organ preservation. This highly effective complex solution, available on the market under the trademarks VIASPAN®, SPS-1® (Organ Recovery Systems, USA), and Belzer UW® Cold Storage Solution (Preservation Solutions Inc., USA), contains a large panel of components, which are aimed at providing an improved viability of organs prior to transplantation [12]. Several reports show its applicability for the hypothermic preservation of MSCs [17, 18]. However, the complex composition of this solution, which needs to be validated, according to clinical GMP standards for injectable devices, complicates its use as a vehicle solution for cell delivery proposes. HypoThermosol® FRS (HTS-FRS, BioLife Solutions, USA) represents another commercially available option for the storage of cells and tissues. The effective preservation has been confirmed for hepatocytes [13, 19], endothelial cells [13], MSCs monolayers [20], and neural stem cells [21]. The HTS-FRS solution is not yet approved by the regulatory authorities for direct administration to patients; nevertheless, several preclinical and clinical trials have already been carried out, using HTS-FRS as a vehicle solution for cell delivery (https://clinicaltrials.gov). It was shown that the application of HTS-FRS for low-volume intravenous injection of allogeneic equine cord blood-derived MSC suspensions was not associated with short-term adverse reactions [22].

Considering the limited number of available possibilities for the cold preservation of cell-based products, the development and optimisation of simple and efficient hypothermic storage solutions specifically for MSCs represents an important issue in regenerative medicine. We built our hypothesis on the idea that different cell types, which have distinct metabolic pathways and membrane transport parameters, would have a different resistance to the crucial factors that appear during the cold storage. It was previously shown that MSCs are more resistant to osmotic stresses, hypoxia, oxidative stress, or even ionizing radiation, compared to more differentiated cells [23–27]. Thus, it was assumed that the combination of the minimal number of essential components within the preservation medium, which are aimed at addressing the most crucial damaging factors that occurred during hypothermia, may be sufficient for maintaining the high viability and functional properties of MSCs and can be easily translated into a clinical-grade manufacturing process as a cell delivery vehicle solution.

In this study, we propose a simple and effective trehalose-based solution for the hypothermic storage of human bone marrow MSC suspensions. We have assessed the ability of this new solution to preserve the viability and functional properties of MSCs and compared its efficiency with other commercially available media.

## 2. Materials and Methods

### 2.1. The Isolation and Expansion of Human Bone Marrow MSCs

The research was approved by the ethical committee of the Institute of Experimental Medicine, Academy of Sciences of the Czech Republic. Upon receipt of the informed consent from donors, who were either subjects in a clinical trial or undergoing orthopaedic surgery, human bone marrow (BM) was obtained from the iliac crest under local or general anaesthesia. To isolate MSCs, BM was applied on Gelofusine R (B. Braun Melsungen AG, Germany) and mononuclear fraction was seeded on plastic flasks (70,000-140,000 cells/cm^2^, TPP Techno Plastic Products AG, Trasadingen, Switzerland) and allowed to adhere [28]. Nonadherent cells were removed after 24 and 48 hours by replacing the media. Adherent cells were cultured at 37°C in a humidified atmosphere containing 5% CO_2_ in MEM Alpha modification medium (Thermo Fisher Scientific Inc., USA, *α*-MEM) containing 5% of pooled platelet lysate (PL, Stemulate®, Cook Regentec, USA) and gentamicin (10 *μ*g/ml; Gentamicin Lek®; Lek Pharmaceuticals, Ljubljana, Slovenia). The medium was changed twice a week. After reaching near-confluence, the cells were harvested by a TrypLE™ solution (Thermo Fisher Scientific Inc., USA). The cells were then seeded again onto a fresh plastic surface at the density 5 × 10^3^ cells/cm^2^. MSCs from passages 2 to 4 have been used in this study.

### 2.2. The Hypothermic Storage of MSCs

The hypothermic storage of hMSC suspensions was prepared in a Buffered Trehalose Solution (BTS, prepared according to Czech Republic patent 306800) [29] and compared to several commercially available media: Ringer's solution for infusion (Baxter Healthcare Ltd.), Plasma-Lyte® 148 (Baxter Healthcare Ltd.), and HypoThermosol® FRS (HTS-FRS, BioLife Solutions Inc.). BTS was prepared from cGMP-grade components (30 mM KH_2_PO_4_, 15 mM Na_2_HPO_4_, 0.5 mM CaCl_2_, 1 mM MgSO_4_, 250 mM trehalose, pH = 7.2, osmolarity = 300 − 350 mOsmol) [29]. Ringer's and Plasma-Lyte® 148 were chosen as commonly used clinical grade vehicle solutions for cell delivery. HTS-FRS was used as a solution, which was reported to be efficient in the hypothermic preservation of tissues and different cell types.

For hypothermic storage, 10^7^ of harvested MSCs were resuspended in 1 mL of corresponding hypothermic storage solution, transferred into 1.8 mL cryovials (Nunc, USA), and placed at 4°C for 24 hrs, 48 hrs, and 72 hrs.

### 2.3. The Cell Viability, Recovery, and Proliferation of MSCs after Hypothermic Storage

The viability of MSCs before and after hypothermic storage was assessed using Trypan Blue plasma membrane integrity assay. Briefly, cell suspension was diluted 1 : 1 with 0.4% Trypan Blue solution (Sigma-Aldrich, USA) in PBS and counted in a Bürker haemocytometer, using standard protocol. The survival rate was expressed as a percentage of viable unstained cells in suspension, in relation to the total number of cells counted in a haemocytometer (*n* = 5).

The recovery of MSCs was assessed by the alamarBlue (AB) test [30, 31], using the same sample before and after hypothermic storage. Briefly, MSCs were resuspended in *α*-MEM, containing 5% PL, plated at a density of 2 × 10^4^ cells/cm^2^ into standard 24-well plates (TPP, Switzerland), and cultured at 37°C, 5% CO_2_, and 95% humidity. The recovery of MSCs was assessed after 24 hrs and 4 days of recultivation. MSCs were incubated for 3 hrs in a culture medium containing 10% AB. The fluorescence level of reduced AB was assessed using a TECAN GENios microplate reader (Tecan Genios; Tecan, Austria) with an excitation wavelength of 550 nm and an emission wavelength of 590 nm. The ratio between fluorescence of experimental and blank sample (without cells) was used as the AB value (*n* = 5). The recovery rate was counted as a percentage ratio between the AB values of cells after cold storage to the same samples before hypothermic preservation. On day 4, the AB values of stored and recultivated MSCs were related to the AB values of the nonstored cells, cultured for the same period of time.

### 2.4. Immunophenotypic Analysis

MSCs were harvested, counted, and characterised by flow cytometry. The cells were characterised by surface markers specific for MSCs [32]: major histocompatibility complex class I (MHC I; Exbio Ltd., Vestec, Czech Republic), CD90 and CD73 (BioLegend, San Diego, CA, USA) and CD105 (Exbio Ltd.), and low expression levels of CD34, HLAD-DR, and CD3 (Beckman Coulter Inc., Brea, CA, USA) and CD45, CD14, CD16, CD19, and CD80 (Exbio Ltd.). All antibodies were firstly titrated and used according to the manufacturer's instructions. Briefly, cell pellets were washed with PBS and after spinning resuspended in PBS again. The cells were incubated in 50 *μ*l of PBS containing specific antibodies for 15 min at room temperature. The cells were then washed with PBS, and 100,000 events were analysed using a FACSCanto II (BD, Franklin Lakes, NJ, USA) flow cytometer. The results were evaluated with FACSDiva Software (BD).

### 2.5. The Differentiation Properties of MSCs

*The in vitro* adipogenic and osteogenic differentiation capacity of MSCs after storage was assessed after seeding of the same amounts (2 × 10^4^/cm^2^) of viable cells into 24-well plates and additional expansion for 2-3 days in *α*-MEM, supplemented with 10% FBS.

*The adipogenic differentiation* medium consisted of *α*-MEM, supplemented with 10% FBS and the following adipogenic stimulants: 0.5 mM 3-isobutyl-1-methylxanthine, 1 *μ*M dexamethasone, 10 *μ*g/ml insulin, and 100 *μ*M indomethacin (all from Sigma-Aldrich, USA). Complete medium changes were performed every 3–4 days. After 21 days of culture with adipogenic supplements, MSCs were fixed in a 4% buffered formalin for 30 min at 4°C and stained with a Nile Red (1 *μ*g/ml in PBS) solution (Sigma-Aldrich, USA) according to the manufacturer's instructions. The cells were assessed using a fluorescent microscope (Nikon, Japan). A quantitative assessment of the Nile Red fluorescence intensity in investigated groups was prepared in a microplate reader (Tecan Genios; Tecan, Austria) at 550 nm/590 nm wavelengths, using a multiple-point reading mode (9 reading points per well). The data were presented as an average Nile Red fluorescence intensity for each condition, after the subtraction of negative control values (obtained from noninduced cells).

*The osteogenic differentiation* medium consisted of *α*-MEM, supplemented with 10% FBS and osteogenic inductors: 0.2 mM ascorbic acid, 10 mM *β*-glycerophosphate, and 1 *μ*M dexamethasone (all from Sigma-Aldrich, USA). Complete medium changes were performed every 3–4 days. Following 21 days of culture, MSCs were fixed in 4% buffered formalin for 30 min at 4°C. Matrix mineralization was assessed using alizarin red (Sigma-Aldrich, USA) staining for 10 min at room temperature. The quantitative analysis of the osteogenic differentiation efficiency was performed by the assessment of calcium accumulation. After a 2 hr extraction of calcium from the cell culture vessels, using 0.1 N HCl, its content was assessed by the standard calcium biochemical assay kit (Erba Lachema Ltd., Czech Republic), according to the manufacturer's instructions. The data were presented as Ca^2+^ concentration (mg/l) after the subtraction of negative control values (obtained from noninduced cells).

### 2.6. The Immunomodulatory Properties of MSCs

The immunomodulatory properties of MSCs before and after 72 hrs of hypothermic storage were assessed by the ability of MSCs to suppress the phytohemagglutinin- (PHA-) induced proliferation of human peripheral blood mononuclear cells (PBMCs) in a direct coculture. PBMCs from at least 3 different human peripheral blood donors (obtained from the Institute of Hematology and Blood Transfusion, Prague, Czech Republic) were isolated by Ficoll-Paque (Miltenyi Biotec Inc., Germany, *ρ* = 1077 g/ml) gradient centrifugation, according to the manufacturer's instructions. For coculture experiments, 2 × 10^4^ viable by Trypan Blue MSCs were seeded into separate wells of 96-well plates and treated with mitomycin C (10 *μ*g/ml) for 2 hrs to arrest the cell proliferation. After 3 washes, MSCs were cultured for 24 hrs before the coculture experiment. Following 24 hrs of culture, the culture medium was discarded and 10^5^ PBMCs were seeded into each well (200 *μ*l/well) in RPMI-1640 medium (Sigma-Aldrich), supplemented with 10% FBS (BIOSERA, France) and PHA (500x, Thermo Fisher Scientific Inc). In each experiment, MSCs from at least 3 donors were cocultured with PBMCs from 3 donors. The final MSC to PBMC ratio comprised of 1 : 5. After 96 hrs of coculture, the proliferation of PBMCs was assessed by AB assay. Briefly, cells in each well were thoroughly resuspended and 100 *μ*l of nonadherent PBMCs were transferred into a fresh well of 96-well plates. 200 *μ*l of 10% AB solution was then added into each well, and the cells were incubated for 4 hrs at 37°C, 5% CO_2_, and 95% humidity. The fluorescence level of the reduced AB was assessed using a TECAN GENios microplate reader (Tecan Genios; Tecan, Austria) with an excitation wavelength of 550 nm and an emission wavelength of 590 nm. The ratio between the fluorescence of experimental and blank samples (without cells) was used as the AB value. The index of PBMC proliferation was determined as a ratio in the AB values of PHA-stimulated (alone or in coculture) and nonstimulated PBMCs.

### 2.7. The Gene Expression Analysis of MSCs after Hypothermic Storage and following TNF-*α*/IFN-*γ* Stimulation

The changes in mRNA expression of the following human genes (IDO-1, PD-L1, IL-6, and GAPDH as a reference gene) were determined in MSC samples before and after 72 hrs of hypothermic storage in HypoThermosol® FRS and BTS preservation solutions. Briefly, MSC suspensions were divided into two parts. One part (around 2-5 × 10^6^ cells) was washed with PBS (1 : 10) and centrifuged at 2000 rpm for 10 min. Cell pellets were stored at -80°C for the following analysis. Additionally, 10^6^ of viable cells were seeded into T75 cell culture flasks (Nunc, USA) in serum-free *α*-MEM, containing an insulin-transferrin-selenium supplement (100x, Thermo Fisher Scientific Inc., USA), antibiotics, 5 ng/ml TNF-*α*, and 5 ng/ml IFN-*γ*. The cells were cultured at 37°C, 5% CO_2_, and 95% humidity for 24 hrs, harvested by trypsinization, washed with PBS, and stored as cell pellets at -80°C for the following analysis. The changes in mRNA expression were determined in samples treated by cytokines by quantitative real-time PCR (qPCR) and plotted against the untreated controls. The RNA was isolated using the RNeasy Mini Kit (Roche, Germany), and RNA amounts were quantified using NanoPhotometer® P 330 (Implen, Germany). The isolated RNA was reverse-transcribed into complementary DNA using the Transcriptor Universal cDNA Master (Roche, Germany) and T100™ Thermal Cycler (Bio-Rad, USA). The qPCR reactions were performed using a cDNA solution, FastStart Universal Probe Master (Roche, Germany), and TaqMan® Gene Expression Assays (Thermo Fisher Scientific Inc., USA). The qPCR was carried out in a final volume of 10 *μ*l containing 65 ng of extracted RNA. The amplification was performed on the real-time PCR cycler (StepOnePlus™, Life Technologies, USA). All amplifications were run under the same cycling conditions: 2 min at 50°C and 10 min at 95°C, followed by 40 cycles of 15 s at 95°C and 1 min at 60°C. All samples were run in duplicate, and a negative control (water) was included in each array. The results were expressed as log2-fold changes of ∆∆Ct values, relative to the control samples untreated by cytokines.

### 2.8. Statistical Analysis

The experiments were performed from MSCs, obtained from 3 different donors in duplicate. The data are presented as mean ± SEM. The statistical analysis was prepared using the Mann-Whitney nonparametric test and considered significant with *p* < 0.05.

## 3. Results

### 3.1. The Viability and Recovery of MSCs after Hypothermic Storage

**Figure 1:**
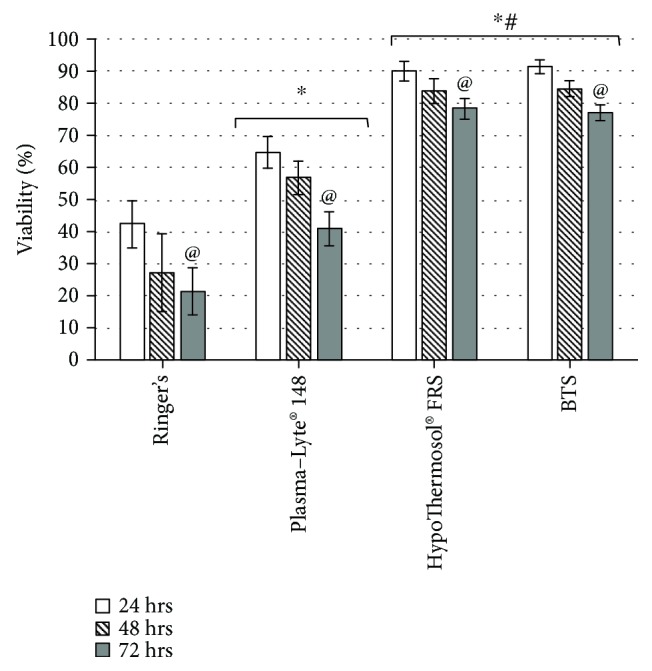
Viability of MSCs after hypothermic storage in different solutions during 24 hrs, 48 hrs, and 72 hrs, determined by Trypan Blue staining. ^∗^Values are significantly higher compared to Ringer's solution (*p* < 0.05); ^#^values are significantly higher compared to Plasma-Lyte® 148 solution (*p* < 0.05); ^@^values are significantly different compared to the same group after 24 hrs of storage (*p* < 0.05).

The viability of MSCs after 24 hrs of storage in Ringer's solution falls dramatically and comprised of 42 ± 8% (Figure 1). The continuous cell storage resulted in a further decrease in cell viability, down to 22 ± 7% at 72 hrs. The viability of MSCs after hypothermic storage in Plasma-Lyte® 148 was significantly higher, compared to Ringers' solution, at each investigated time point. After 72 hrs of storage in this solution, the viability of MSCs comprised of 41 ± 5% (Figure 1).

In contrast, the viability of MSCs after hypothermic storage in BTS or HTS-FRS was preserved after 72 hrs at significantly higher levels. The values were not significantly different between the groups and comprised of 78 ± 3% and 77 ± 2% for the HTS-FRS and BTS groups correspondingly (Figure 1).

**Figure 2:**
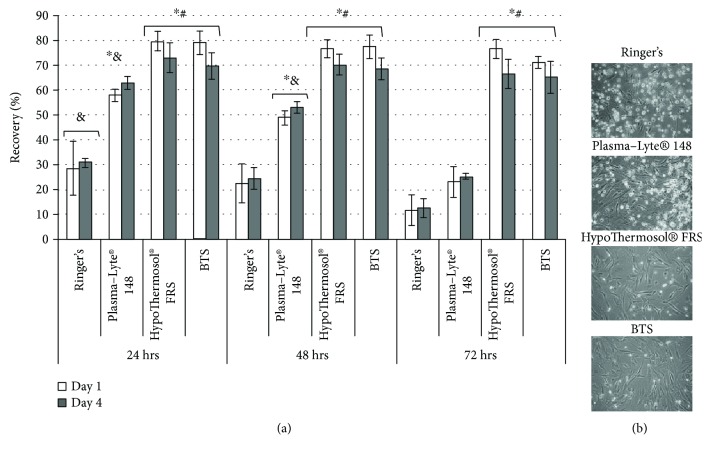
(a) Recovery of MSCs after hypothermic storage in different solutions and following recultivation for 1 and 4 days (alamarBlue assay). (b) Phase-contrast microscopy of MSCs after cold storage and 24 hrs of recultivation. ^∗^Values are significantly higher compared to Ringer's solution (*p* < 0.05); ^#^values are significantly higher compared to Plasma-Lyte® 148 solution (*p* < 0.05); ^&^values are significantly different compared to the same group after 72 hrs of storage (*p* < 0.05).

The same situation was observed after the determination of cell recovery; however, the values were lower, compared to the viability (Figure 2).

The lowest cell recovery was obtained after 48 hrs and 72 hrs of storage in Ringer's solution (23 ± 7% and 11 ± 8%, correspondingly). The recovery of MSCs stored for 48 hrs in Plasma-Lyte® 148, although being significantly higher than in Ringer's solution, comprised of only 49 ± 3% and decreased to 23 ± 2% after 72 hrs. At the same time, the cold storage in BTS or HTS-FRS resulted in a lower decrease in MSC recovery. After 24 hrs of preservation, the recovery rate comprised of about 80%, while after 72 hrs of storage the values were 74 ± 2% and 71 ± 3% correspondingly for the BTS and HTS-FRS groups (Figure 2(a)). The morphology of MSCs after 72 hrs of hypothermic storage and subsequent culture during 24 hrs was in contrast in the BTS and HTS-FRS groups, compared to Ringer's and Plasma-Lyte® 148 (Figure 2(b)). The preservation of fibroblast-like morphology was revealed after storage in the BTS and HTS-FRS, while in Ringer's solution and Plasma-Lyte® 148, a large amount of cellular abnormalities together with a high number of unattached cells were found (Figure 2(b)). The capacity of MSCs to proliferate in vitro was preserved after hypothermic storage in all of the studied solutions on a similar level. Therefore, the recovery rate at day 4, determined as a ratio to the control (fresh) group, was the highest after hypothermic storage in the BTS and HTS-FRS (Figure 2(a)).

The presented data show that the hypothermic preservation of MSCs during 24 hrs–72 hrs, which is needed to conduct all of the necessary microbiological quality control tests, is detrimental for cells when using conventional saline solutions (e.g., Ringer's or Plasma-Lyte® 148). However, the utilization of the BTS or HTS-FRS for cell storage provides high (more than 70%) viability and cell recovery, even after 72 hrs of hypothermic preservation.

### 3.2. The Immunophenotype of MSCs after Hypothermic Storage

To analyse whether the hypothermic storage conditions would affect the identity of MSCs, we studied the specific immunophenotype (Table 1) of MSCs after cold preservation in different conditions. The immunophenotypic analysis revealed that, independently of the duration of hypothermic preservation at 4°C and applied storage media, viable MSCs preserved their specific immunophenotype. More than 95% of viable cells had the immunophenotype CD105+/CD90+/CD73+/CD34-/CD45-/CD19-/CD14-/HLA-DR- which corresponded with the minimal criteria for multipotent mesenchymal stromal cells [32].

### 3.3. The Differentiation Properties of MSCs after Hypothermic Storage

**Figure 3:**
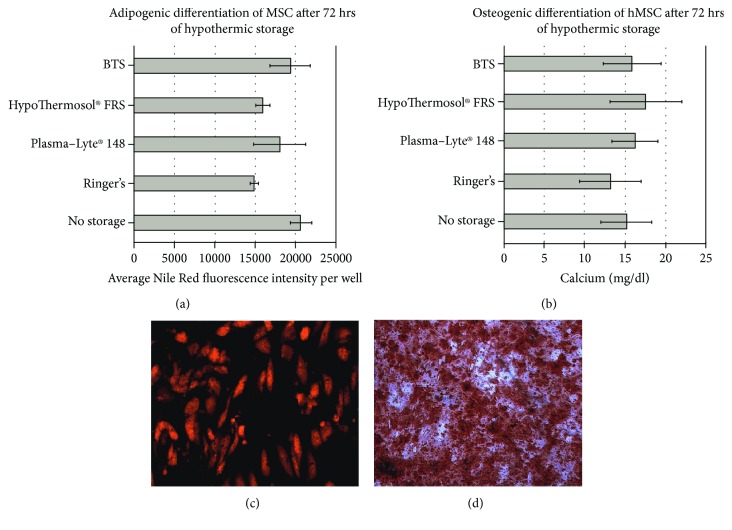
Adipogenic (a, c) and osteogenic (b, d) differentiation of human MSCs after 72 hrs of hypothermic storage in different solutions and following recultivation. (a) Average Nile Red fluorescence intensity; (b) accumulated calcium content; (c) Nile red staining ×100; (d) alizarin red staining, ×100.

The following assessment of the MSC capacity for induced differentiation towards adipogenic and osteogenic lineages was prepared by seeding the same amounts of viable MSCs and the following culture in differentiation media (Figure 3). The quantitative analysis of the differentiation efficiency did not reveal any significant differences between the applied hypothermic storage conditions, confirming the ability of MSCs to recover during prolonged *in vitro* culture (Figures 3(a) and 3(b)). Independently of the hypothermic storage conditions, the in vitro cell culture in the presence of specific inducers led to the accumulation of intracellular lipid droplets in adipogenic cultures (Figure 3(c)) and the appearance of a calcified matrix in osteogenic cultures, positively stained by alizarin red (Figure 3(d)). Chondrogenic differentiation was impaired after 72 hrs of hypothermic storage in Ringer's solution and Plasma-Lyte® 148. In this case, no pellets were able to be formed due to the initially poor recovery of MSCs, stored in these conditions. This necessitates the additional expansion of MSCs after storage in saline solutions prior to application.

### 3.4. The Immunomodulatory Properties of MSCs after Hypothermic Storage

**Figure 4:**
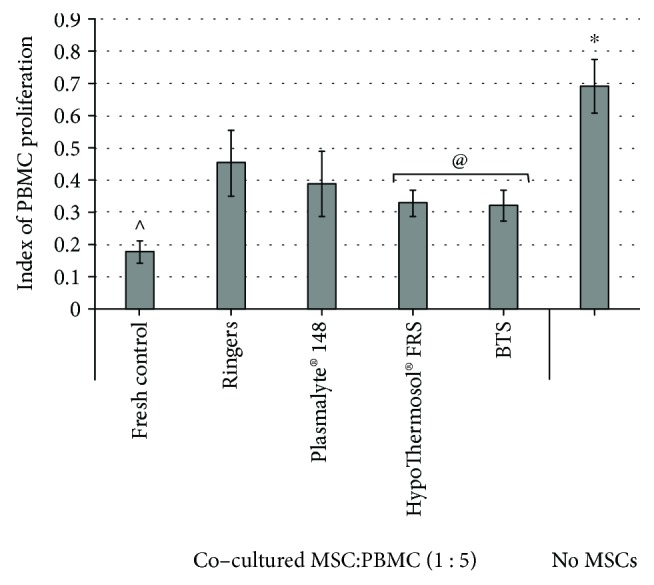
Proliferation of PHA-stimulated PBMC in coculture with MSCs before and after 72 hrs hypothermic storage in different preservation solutions. ^∗^Data is significantly different compared to MSC: PBMC coculture groups (*p* < 0.05); ^@^data is significantly different compared to Ringer's solution (*p* < 0.05); ^^^values are significantly different compared to all coculture groups after 72 hrs of storage (*p* < 0.05).

To reveal whether hypothermically stored MSCs preserved their immunomodulatory capacity, the cells were cocultured during 96 hrs with PHA-stimulated PBMCs. As shown in Figure 4, MSCs before and after 72 hrs of hypothermic storage in different preservation solutions were able to significantly suppress the PHA-induced proliferation of PBMCs in the direct coculture. Although the nonstored MSCs (fresh control) had a stronger influence on PBMC proliferation, the inhibitory activity of hypothermically stored MSCs had a similar tendency to the recovery rate, as previously observed. The most pronounced PBMC growth suppression was observed in the cocultures with MSCs, stored in BTS or HTS-FRS (Figure 4), being significantly stronger than the MSCs kept in Ringer's solution.

**Figure 5:**
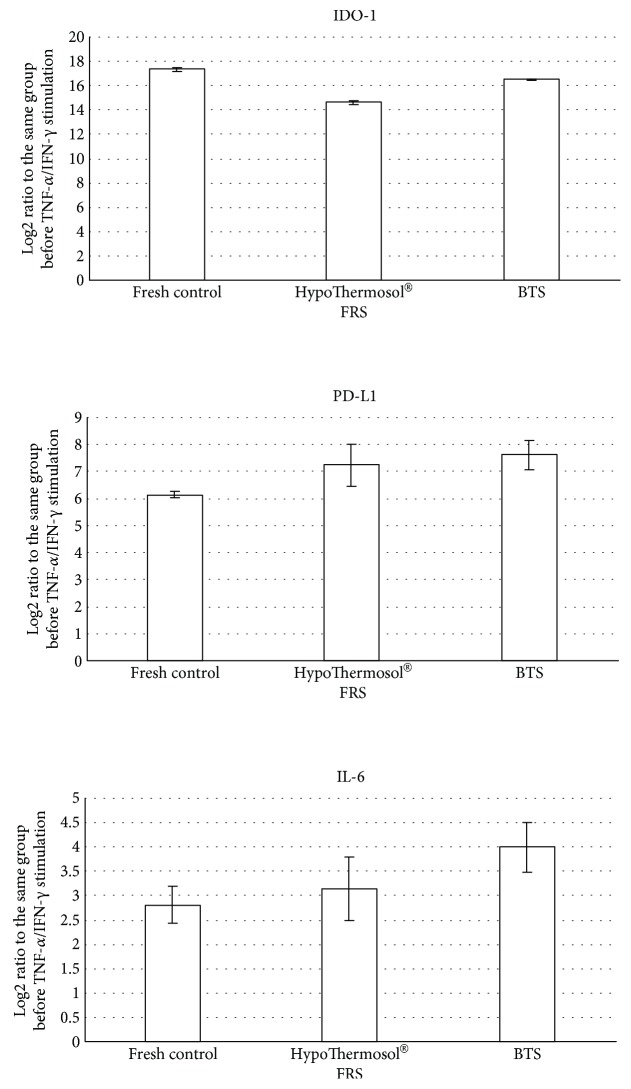
Gene expression analysis of MSCs after 72 hrs of hypothermic storage and following TNF-*α*/IFN-*γ* stimulation.

The ability of stored MSCs to respond to inflammatory priming (IFN-*γ* and TNF-*α* stimulation) is another aspect, showing the therapeutic potential of MSCs prior to clinical application [33, 34]. In this study, the expression of genes involved in MSC immunomodulation (IDO-1, PD-L1, and IL-6) was assessed immediately after the storage in BTS and HTS-FRS (chosen as the most effective preserving media) and after 24 hrs *in vitro* stimulation with cytokines (Figure 5). Nonstored MSCs served as the control.

The levels of IDO-1, PD-L1, and IL-6 expression were significantly increased after IFN-*γ* and TNF-*α* stimulation confirming the preservation of the functional activity of MSCs after hypothermic storage. There were no significant differences in the response to proinflammatory factor stimulation between the nonpreserved cells and those after 72 hrs of hypothermic storage (Figure 5).

## 4. Discussion

In this study, we have compared the efficiency of the developed BTS with other commercially available solutions, regarding their ability to preserve the viability and functional properties of MSC suspensions during hypothermic storage. We tried to simulate real conditions, applied in biotechnological laboratories during the manufacturing of clinical-grade MSC-based products, and their preparation for further administration to the patient. To conduct the essential quality control analysis, the finally prepared MSC suspensions should be stored in a vehicle solution, which can be directly administered to a patient and which also provides the necessary protection to maintain the viability and identity of the cells.

We have assessed whether two commonly used saline solutions for injections (Ringer's solution and Plasma-Lyte® 148) are able to provide the maintenance of essential cell functions during 1-3 days of hypothermic storage at 2-8°C. It was discovered that in contrast to Ringer's solution, Plasma-Lyte® 148 can provide the survival of MSCs at about a 50% level after 48 hrs of storage; however, after 72 hrs of cold preservation, the recovery of cells was not higher than 25%. Nevertheless, the viable cells preserved their specific immunophenotype and capability for induced differentiation into adipogenic and osteogenic lineages. Previously, Chen et al. showed that cold (2-8°C) preservation in a 0.9% sodium chloride and Plasma-Lyte A solution already results in more than a 25% decrease in MSC attachment after 6 hrs of storage [35]. However, these values were higher compared to the same duration of storage at room temperature. Similar results have been shown by Pal et al., where the viability of MSCs after 8 hrs of storage at 4°C decreased to about 15% in Plasma-Lyte A, 0.9% saline solution, or Dulbecco's phosphate-buffered saline [36]. The low efficiency of saline-based solutions, which were not specially formulated to preserve the viability of cells during hypothermic preservation, is also confirmed by several studies showing the cold storage of MSCs in a complete culture medium [37, 38]. Pogozhykh et al. showed less than 50% viability of placenta-derived MSCs after 48–72 hrs of storage in a culture medium at 4°C, additionally accompanied by a huge (5-10 times) drop of cell numbers, compared to the nonstored control [37]. Swioklo et al. showed only a 20% viable adipose tissue of MSC recovery after 72 hrs of hypothermic storage in a culture medium [38]. The low viability was also shown for MSCs stored at 4°C in the adherent state [18]. The authors could not detect any viable MSCs after 48 hrs of hypothermic preservation, either in a culture medium or in Hank's balanced salt solution [18].

The change in cell ionic homeostasis which occurred under hypothermic conditions has been confirmed by many authors and thoroughly reviewed [11]. Hypothermic storage leads to a K^+^ loss from cells and is simultaneously accompanied by the intracellular diffusion of Na^+^ and Cl^−^, leading to a strong volume increase and cell swelling. Thus, one of the main reasons for low cell viability in standard saline-based solutions is more likely associated with the high amounts of sodium and chlorine ions in the preservation medium. Therefore, even these saline solutions are undoubtedly an optimal choice for the injection of fresh (nonstored) cells; they possess poor preservation potential during hypothermic storage. To reduce the cold-induced cell swelling, the partial substitution of these ions by nonpermeable, osmotically active components (e.g., saccharides) is a common option applied during the development of specialised hypothermic storage solutions [11, 12, 16, 39, 40].

In our study, we developed a simple solution for the hypothermic storage of MSCs. Considering the high resistance of MSCs to various stress factors, we built our hypothesis on the basis that the essential functions of MSCs can be preserved using a simpler composition of the solution, providing stability of pH, close to physiological osmolarity, and preventing abnormal ion exchange and cell swelling. Such a hypothesis also seemed promising from a translational point of view: since all the components used for the preparation of the solution should be validated and each one may provide a negative action in vivo, the fewer number of chemicals in the mixture may simplify the use of such a medium as a vehicle solution for cell delivery. Previously, several modifications of different simple solutions, containing varying concentrations of sucrose and/or high molecular weight polyethylene glycol, BES, or other additives, have been shown to have a positive effect on the viability of hepatocytes [39, 41, 42], kidney cells [43], and endothelial cells [16] at cold storage conditions.

We have compared the efficiency of BTS with HypoThermosol® FRS, since this preservation medium was shown to have a confirmed positive impact on the survival of adherent MSCs [20]. According to available information [13, 44], the composition of a HypoThermosol® FRS solution is comprised of a specially formulated ionic mixture, pH buffers (including HEPES), several nonpermeable substances (sucrose, lactobionate, and mannitol), and adenosine and glutathione as metabolites. Overall, this composition provides the necessary defence for different cell types during hypothermic storage at 2-8°C [13, 19, 21]. We have determined that the preservation capacity of BTS is similar to HTS-FRS, since no significant differences were observed between these two solutions in all our experiments. Both solutions provided more than 70% recovery of MSCs, which could proliferate, differentiate, and suppress the proliferation of PBMCs in a coculture and respond to a proinflammatory cytokine stimulation in vitro, confirming the preservation of their therapeutically associated properties.

The BTS proposed in our study contains only 5 components, including inorganic pH buffers, and only one nonpermeable substance (trehalose) as a stabilising agent, providing balance in ion transport concentrations and protecting the cells against critical swelling. We chose trehalose in our study due to its confirmed ability to stabilise the phospholipid bilayers and labile proteins in mammalian cells [45–48] as well as reduce oxidative stress [49]. Trehalose is highly employed in cryopreservation studies as a nontoxic additive, capable of significantly reducing the concentration of permeable cryoprotectants (e.g., Me_2_SO) or even excluding it from the freezing medium [2, 50, 51]. The positive effect of trehalose has been shown in ophthalmological studies to improve corneal regeneration after acute corneal damage [52] or dry eye syndrome [53]. We have previously shown that trehalose is a very potent therapeutic agent in corneal regeneration, which favourably influences the oxidative damage of the cornea and suppresses proinflammatory cytokine production [49, 52, 54]. Moreover, trehalose exhibits strong neuroprotective properties by stabilising proteins and significantly promoting autophagy [55, 56]. Recently, trehalose 90 mg/ml intravenous solution has received orphan drug designation from the Committee for Orphan Medicinal Products for treatment of spinocerebellar ataxia [57]. Moreover, the presence of trehalose in several commercially available products for topical (Thealoz®, Laboratoires Théa, Clermont-Ferrand, France) or injectable pharmaceutical therapeutics (e.g., Avastin®, Advate®, or Lucentis®) is promising for the future potential of using BTS or its modification as a vehicle solution for cell administration.

## 5. Conclusions

In this study, we have demonstrated the application of a simple buffered trehalose solution for the hypothermic storage of MSC suspensions with a high potential of translation into clinical practice. We found that the conventional vehicle solutions for cell administration cannot provide suitable viability of MSCs after hypothermic storage for 48-72 hrs, which may lead to nonsufficient therapeutic outcomes of MSC-based products in clinical trials. Subsequently, the BTS is capable of maintaining the viability and functional activity of MSCs for up to 3 days, which allows for the conduct of quality control analysis or transport prior to cell administration. The next stages of the study will be directed at preclinical and clinical studies, with the aim of confirming the safety of using BTS as a vehicle solution for cell delivery.

## Figures and Tables

**Table 1:** The percentage of cells with the immunophenotype CD105+/CD90+/CD73+/CD34-/CD45-/CD19-/CD14-/HLA-DR- after hypothermic storage in different preservation solutions.

Preservation solutions	The duration of hypothermic storage		
	24 hrs	48 hrs	72 hrs
Ringer's	94.5%	94.7%	97.4%
Plasma-Lyte® 148	93.7%	95.8%	97.1%
HTS-FRS	93.5%	95.2%	94.3%
BTS	96.2%	92.8%	98.3%

## Data Availability

The data used to support the findings of this study are currently under embargo while the PCT patent application is under consideration. Requests for data, 12 months after publication of this article, will be considered by the corresponding author.
